# Health inequalities among young workers: the mediating role of working conditions and company characteristics

**DOI:** 10.1007/s00420-023-02010-6

**Published:** 2023-10-09

**Authors:** Marvin Reuter, Claudia R. Pischke, Mariann Rigo, Katharina Diehl, Jacob Spallek, Matthias Richter, Claudia Hövener, Nico Dragano

**Affiliations:** 1 Junior Professorship for Sociology, esp. Work and Health, Department of Sociology, University of Bamberg, Feldkirchenstraße 21, 96045 Bamberg, Germany; 2 Institute of Medical Sociology, Centre for Health and Society, Medical Faculty and University Hospital Düsseldorf, Heinrich-Heine-University Duesseldorf, 40225 Duesseldorf, Germany; 3 Department of Medical Informatics, Biometry and Epidemiology, Chair of Epidemiology and Public Health, Friedrich-Alexander-Universität Erlangen-Nürnberg (FAU), 91054 Erlangen, Germany; 4 Department of Public Health, Brandenburg University Cottbus-Senftenberg, 01968 Senftenberg, Germany; 5 Department of Sport and Health Science, Technical University of Munich, 80992 Munich, Germany; 6 Unit of Social Determinants of Health, Robert Koch Institute, Nordufer 20, 13353 Berlin, Germany; 7 Lausitz Center for Digital Public Health, Brandenburg University of Technology Cottbus-Senftenberg, 01968 Senftenberg, Germany

**Keywords:** Health inequalities, Young workers, Mediation, Job demands, Company level, Socio-economic position

## Abstract

**Objective:**

Few studies have investigated health inequalities among young workers. The objectives of this study are to assess the extent of health inequalities in a sample of job starters and to explore the contribution of job demands and organisational factors.

**Methods:**

We analyze data from the BIBB/BAuA Youth Employment Survey 2012. The cross-sectional survey includes a representative sample of 3214 German employees, apprentices, and trainees aged 15–24 years. Individuals were grouped by their years of schooling into low (< 12 years) and high levels of education (≥ 12 years). Regression analysis estimated the link between education and four health outcomes: self-rated health, number of health events, musculoskeletal symptoms, and mental health problems over the last 12 months. Counterfactual mediation analysis tested for indirect effects of education via working conditions (i.e., physical and psychosocial job demands) and company characteristics (i.e., company size, health prevention measures, financial situation, downsizing). All analyses were adjusted for age, sex, nationality, region, working hours, job tenure, employment relationship, and economic sector.

**Results:**

Highly educated workers reported better self-rated health (*b* = 0.24, 95% CI 0.18–0.31) and lower numbers of health events (Rate Ratio (RR) = 0.74, 95% CI 0.67–0.82), musculoskeletal symptoms (RR = 0.73, 95% CI 0.66–0.80) and mental health problems (RR = 0.84, 95% CI 0.76–0.93). Total job demands explained between 21.6% and 87.2% of the educational differences (depending on health outcome). Unfavourable company characteristics were associated with worse health, but showed no or only small mediation effects.

**Conclusions:**

Health inequalities are already present at the early working career due to socio-economically stratified working hazards. To enhance prevention measures that aim at reducing inequalities in workplace health, we propose shifting attention towards earlier stages of life.

**Supplementary Information:**

The online version contains supplementary material available at 10.1007/s00420-023-02010-6.

## Introduction

In ageing societies worldwide, healthy working conditions are a prerequisite for expanding labour force participation and meeting the challenges of demographic change. However, past research has shown that workers in lower socio-economic positions—referring to persons with lower levels of education, prestige or income—carry greater risks for poor health. This association has been observed across various health indicators such as self-rated health (Mackenbach et al. 2008), musculoskeletal diseases (Karran et al. 2020), mental health problems (Muntaner et al. 2004; Lund et al. 2018), as well as occupational injuries and accidents (Khanzode et al. 2012). As a consequence, socio-economic disadvantage was found to be associated with elevated rates of sickness absence (Hansen and Andersen 2008; Christensen et al. 2008), higher probabilities for disability pension (Perhoniemi et al. 2020), and premature transitions from work to retirement (Fisher et al. 2016) in several countries.

Socio-economic inequalities in health are driven by multiple aspects (including behavioural and material factors), but occupational exposures seem to be important as well (Moor and Spallek 2017). The mediation hypothesis assumes that socio-economically disadvantaged workers are more likely to be exposed to dangerous or unhealthy working conditions, which, in turn, cause disparate health risks (Clougherty et al. 2010). At the individual-level, this concerns the performance of different job tasks with certain physical and psychosocial demands (Yaris et al. 2020). Physical demands can arise from ergonomic hazards (e.g., lifting and carrying heavy loads) or environmental risks (e.g., exposure to hazardous substances, dusts or gases). Psychosocial demands include aspects of the work organisation (e.g., working long hours or shifts), psychological factors (e.g., working under time pressure), decision latitude (e.g., having control over the amount of work) or the social environment (e.g., receiving support from colleagues). Additionally, located at the meso-level, unfavourable company characteristics might also play a role, for instance, a lack of safety and health measures, experiences of insecurity arising from organisational downsizing or a poor economic situation of the company (Landsbergis et al. 2014).

A first systematic literature review considering the mediation hypothesis was conducted in 2013 based on 17 longitudinal studies (Hoven and Siegrist 2013). The included studies mostly demonstrated that the association between socio-economic position and health was attenuated when simultaneously controlling for job demands. A second review was based on 20 cross-sectional and longitudinal studies focussing on self-rated health as the outcome variable (Dieker et al. 2019). Accordingly, the review found that physical and psychosocial job demands explained around one-third of the association between socio-economic position and self-rated health.

Available studies generally support the mediation hypothesis, but share important limitations. First, previous studies only focussed on job demands as individual-level mediators, but company characteristics at the meso-level have not received much attention, yet. Second, research thus far strongly focussed on middle- and older-age workers. However, we theorise that the first years on the job might be even more important. In a life course perspective, health inequalities established early in life are highly problematic, because they often persist and even exacerbate over the remaining life course (Ben-Shlomo and Kuh 2002; Ferraro et al. 2009). This might be particularly true for the time from late adolescence to young adulthood (15–24 years), where individuals complete formal school education and transition to the labour market (Reuter et al. 2022). If health inequalities are already present among young adults entering their first job, prevention strategies could be more effective when also being implemented in these early life stages. However, evidence for the extent and causes of occupational health inequalities in this population is scarce. Two studies from Canada and Finland suggest that young workers—although being generally in good health—already show pronounced socio-economic variation with regard to self-rated health and sickness absence (Karmakar and Breslin 2008; Sumanen et al. 2015). However, mechanisms for these inequalities remain unclear.

This paper aims to extend knowledge about occupational health inequalities in a representative sample of German workers between the ages of 15–24 years. Specifically, the study has two objectives. First, the study aims to investigate the association between education and health among young workers. The second purpose is to assess the extent to which job demands and company characteristics explain health inequalities among young workers. By examining both individual-level (job demands) and meso-level (company characteristics) factors, an opportunity arises for a comparative analysis.

## Methods

### Data

We use data of the BIBB/BAuA Youth Employment Survey 2012 (*Jugenderwerbstätigenbefragung*, J-ETB), a cross-sectional study conducted by the Federal Institute for Vocational Education and Training (BIBB) and the German Federal Institute for Occupational Safety and Health (BAuA) (Schmiederer 2015). The J-ETB is a representative sample of employees, apprentices, and trainees between the ages of 15 and 24 years, who work or are currently in training for at least 10 h per week. Study participants were selected based on landline and mobile phone numbers (dual framing approach) following a multi-stage sampling process, in which a random sample of households was drawn and individuals in these households were selected. Individuals were not interviewed, if they only reported volunteer work or if they did not speak German sufficiently. Computer-Assisted Telephone Interviews (CATI) were carried out from October 2011 to March 2012 by trained interviewers. The response quote among eligible persons was 48.3%.

### Study sample

The initial sample included 3214 workers. We excluded 56 participants reporting more than 70 days of sickness during the past year to avoid bias resulting from chronic health problems. Furthermore, we excluded 16 solo self-employed because company characteristics were not assessed in this group. Finally, the study sample used for the following analyses comprises of 3142 workers.

### Variables

#### Health measures

We used four health indicators. First, self-rated health was assessed by asking the question “How would you describe your general state of health?” followed by a five-point Likert response format (poor, less good, good, very good, excellent). Second, we assessed the number of days with a health event in the past year by combining the number of self-reported sickness absence and sickness presence days by building a sum score (Gerich 2015):

$$\text{Health events}=\text{Sickness absence}+\text{sickness presence}$$

This approach takes into account that workers differ in their likelihood to opt for presenteeism, which is continuing to work despite being ill or feeling unwell (Reuter et al. 2021). Third, participants were presented a list of seven symptoms of musculoskeletal disorders (i.e., pain in back, neck/shoulder, arms, hand, hips, knees, legs/feet) and five symptoms of mental health problems (i.e., sleep disturbances, tiredness/faintness/fatigue, nervousness/irritability, low mood, emotional exhaustion) and they were asked to indicate whether they experienced each of them during work or working days in the past 12 months (a list is to find in the Appendix, e-Table 1). We constructed a sum score for symptoms of musculoskeletal disorders (7 items, Cronbach’s alpha = 0.68) and a sum score for symptoms of mental disorders (5 items, Cronbach’s alpha = 0.72).

#### School education

The level of school education was used as an indicator of respondents’ socio-economic position. We did not consider vocational or university degrees because parts of young workers were still in training, while school education was generally completed when being surveyed. We calculated a variable indicating the years of education equivalent to the school-leaving certificate (range 9–13) and distinguished between low (< 12 years) and high education (≥ 12 years), dividing between lower and higher secondary school education. As some individuals younger than 18 years were not yet able to graduate from higher secondary school, we controlled for this possible selection bias by adjusting for participant’s age in all multivariable analyses (restricting the sample to participants aged 18 years or older yielded identical results).

#### Job demands

Job demands at the individual-level were assessed through a validated questionnaire comprised of 41 indicators related to six dimensions (ergonomic demands, environmental demands, social support, decision latitude, psychological demands, working time demands) (Kroll 2011; Meyer and Siefer 2021). Ordering of indicators was randomised. We counted the number of demands that workers stated to experience “frequently” during the course of their work (versus “sometimes”, “rarely”, or “never”). For positive items (social support and decision latitude), we counted the absence of a job resource by dichotomising between “never” versus other responses (“rarely”, “sometimes”, “frequently”). We constructed a subscale of physical demands through four indicators of ergonomic and 10 indicators environmental demands (range 0–14, Cronbach’s alpha = 0.81). Psychosocial demands were assessed through six indicators of (low) social support, three indicators of (low) decision latitude, 11 indicators of psychological demands, and seven indicators of working time demands (range 0–21, Cronbach’s alpha 0.64). An overall sum score for the 41 indicators of job demands yielded good internal consistence (Cronbach’s alpha = 0.78). A complete list of job demands and the Cronbach’s alpha for each subscale can be found in the Appendix (e-Table 2).

#### Company characteristics

Company characteristics at the meso-level were the company size (number of persons employed in the enterprise), economic situation of the company (assessed as “less than good” or “poor” by the employee or the company owner), whether the company introduced health promotion measures within the past two years, and whether downsizing measures had been carried out in the company within the two past years (staff reduction or dismissals).

#### Control variables

To adjust for socio-structural differences between workers with a low versus a high level of education, as well as to control for possible confounding, we considered socio-demographic (age, sex, nationality, region) and work-related covariates (employment relation, weekly working hours, job tenure, economic sector) in all multivariable analyses (described in more detail under “Statistical analysis”). Region is a variable storing information about the workplace location (East Germany, West Germany). The employment relation is a variable describing an individual’s formal labour status (employee, self-employed, apprenticeship or student job). The company’s economic sector was based on the German Classification of Economic Branches 2008 (Klassifikation der Wirtschaftszweige 2008). Working time was used as a variable controlling for variations in exposure time. We considered the contractual working hours for employees and the actual working hours for those who were self-employed. We categorised individuals as either full-time or part-time workers based on the European Labor Force Survey’s definition of part-time work, which refers to a weekly working time below 30 h (OECD 2020). By employing this approach, we mitigated any potential overlap with the “long working hours” indicator used to assess psychosocial job demands.

#### Missing information

Patterns of missing values in variables of interest are described in the Appendix (e-Table 3). The occurrence of missing values for variables related to health and job demands was minimal, ranging from 0.0% to 2.2%. However, there were slightly higher proportions of missing values observed in the company characteristics variables, ranging from 2.7% to 13.8%. A complete case analysis would result in the loss of 761 observations (23.7%). Little’s MCAR test was positive (Chi-square(1066) = 1751.9, *p* < 0.001), thus, we decided to impute missing information using chained equations with a predictive mean matching procedure. A comparison of the original versus the imputed data set can be found in the Appendix (e-Table 4).

### Statistical analysis

First, descriptive statistics for all variables by levels of education are presented in Table 1. Second, we studied the association between education and health, as well as between potential mediators and health, each adjusted for control variables in Table 2. Linear regression analysis was used for self-rated health and modified Poisson regression with robust variance estimation for outcomes with count event character (health events, number of musculoskeletal symptoms, number of mental health problems). As to be expected, count variables were skewed and zero-inflated (see Supplementary information, e-Fig. 1). Poisson regression accounts for non-normality of the data while robust variance estimation corrects for heteroscedasticity of residuals (Cameron and Trivedi 2013). Regression estimates were expressed as non-standardised linear regression coefficients for self-rated health and Rate Ratios (RR) for count variables. The RRs indicate the relative difference in the average number of events or symptoms between workers with high and low levels of education. Furthermore, we illustrate the frequency of each of the 41 indicators of physical and psychosocial job demands by the level of education adjusted for covariates. Therefore, we converted regression estimates obtained from Poisson models by a post-estimation command into average adjusted predictions (AAPs) and plotted them along with respective 95% confidence intervals (CI) in Fig. 2 (Williams 2012).

**Fig. 1:**
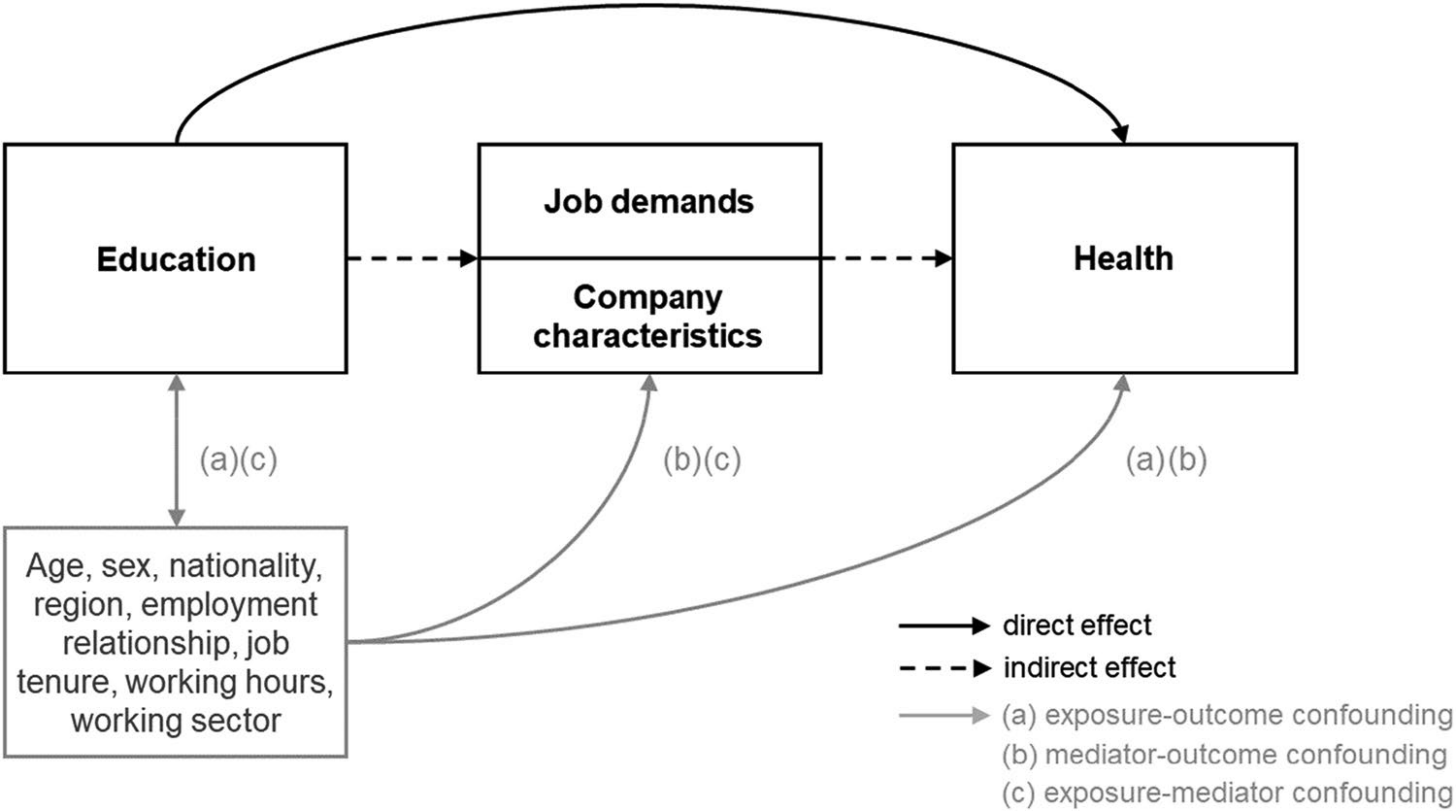
Mediator model illustrating the relationship between education, health and the mediator variables (job demands and company characteristics)

Third, we performed simulation-based mediation analysis within the counterfactual framework to evaluate the role of job demands and company characteristics for health inequalities (Imai et al. 2010). We disentangled the total effect of high versus low education on each health outcome into an indirect effect that went through a mediator and a direct effect that was independent of a mediator (Baron and Kenny 1986). We also controlled for confounding in the relationship between education and health (exposure-outcome confounding), education and job demands or company characteristics (exposure-mediator confounding), and job demands or company characteristics and health (mediator-outcome confounding) (see Fig. 1) (VanderWeele 2016). In addition to socio-demographic characteristics (sex, age, nationality and workplace region), we hold working sectors constant across different levels of education to account for selection processes (i.e., working sectors expose to different levels of physical and psychosocial job tasks) and controlled for different exposure time of job demands by adjusting for working hours, job tenure and the employment relation (full-time jobs, apprenticeships or student jobs).

Mediation analysis was carried out with Stata’s “mediate” package (Hicks and Tingley 2011). Bootstrapping with 1000 repetitions was used to calculate confidence intervals for effect estimates. A significant indirect effect, indicated by a confidence interval that does not overlap “0”, is used as the criteria for assessing presence of mediation. Furthermore, the percentage mediated was calculated by dividing the indirect effect by the total effect. All analyses were performed using Stata 16.1 MP (64-bit, StataCorp LLC, College Station, TX, USA).

## Results

### Descriptive statistics

The distribution of the variables of interest by participants’ level of education is shown in Table 1. Overall, respondents with a high level of education reported more favourable health (despite in terms of mental health problems). Furthermore, workers with a high level of education reported fewer physical and psychosocial job demands and worked in larger companies, but differed not according to other company characteristics. Furthermore, better educated workers were older, more often female, had more often German nationality, were more often from East Germany, had fewer working hours, lower job tenure, worked more often in student jobs, and less often in manufacturing, industry, or construction.

**Table 1:** Descriptive statistics by respondents’ school education

		Low level of education (< 12 years)		High level of education (≥ 12 years)		
Variable	Categories (or range)	N (Col%)	Mean (SD)	N (Col%)	Mean (SD)	*p* value
Health outcomes						
Self-rated health	(1 = poor to 5 = excellent)		3.6 (0.8)		3.8 (0.8)	< 0.001
Number of health events	(0–70)		10.6 (12.5)		7.7 (10.1)	< 0.001
Number of musculoskeletal symptoms	(0–7)		1.7 (1.7)		1.2 (1.4)	< 0.001
Number of mental health problems	(0–5)		1.1 (1.4)		1.1 (1.4)	0.161
Job demands						
Physical demands	(0–14)		4.0 (3.0)		2.1 (2.5)	< 0.001
Psychosocial demands	(0–21)		4.6 (2.9)		4.0 (2.7)	< 0.001
Total job demands	(0–27)		8.5 (4.8)		6.1 (4.3)	< 0.001
Company characteristics						
Company size	(0– ≥ 1000)		227.4 (333.5)		296.8 (376.7)	< 0.001
Workplace health promotion measures	No	1049 (55.5)		687 (54.8)		0.698
	Yes	840 (44.5)		566 (45.2)		
Economic situation of the company	Good or very good	1798 (95.2)		1186 (94.7)		0.506
	Less than good or poor	91 (4.8)		67 (5.3)		
Downsizing in the past two years	No	1311 (69.4)		891 (71.1)		0.306
	Yes	578 (30.6)		362 (28.9)		
Control variables						
Age in years	(15–24)		20.2 (2.3)		21.6 (1.8)	< 0.001
Sex	Males	1141 (60.4)		587 (46.8)		< 0.001
	Females	748 (39.6)		666 (53.2)		
Nationality	German	1786 (94.5)		1210 (96.6)		0.008
	Non-German	103 (5.5)		43 (3.4)		
Region	West Germany	1618 (85.7)		998 (79.6)		< 0.001
	East Germany	271 (14.3)		255 (20.4)		
Weekly working hours	Part-time (< 30 h)	257 (13.6)		381 (30.4)		< 0.001
	Full-time (≥ 30 h)	1632 (86.4)		872 (69.6)		
Job tenure in years	(0–11)		2.1 (1.8)		1.8 (1.5)	< 0.001
Employment relation	Employee	846 (44.8)		384 (30.6)		< 0.001
	Self-employed	19 (1.0)		11 (0.9)		
	Apprenticeship	851 (45.1)		447 (35.7)		
	Student job	173 (9.2)		411 (32.8)		
Economic sector	Agriculture, mining, energy, water	47 (2.5)		30 (2.4)		< 0.001
	Manufacturing, industry, construction	783 (41.5)		266 (21.2)		
	Finance, business, personal services	680 (36.0)		580 (46.3)		
	Public services and health	379 (20.1)		377 (30.1)		
Total		1,889 (100.0)		1,253 (100.0)		

Data: BIBB/BAuA Youth Employment Survey 2012. *n* = 3142. Significance test for group differences by a chi-squared (categorical variable) or student’s *t* test (continuous variable)

*SD* standard deviation, *Col*% column percentage

### Multivariable statistics

In Table 2, multivariable regression analyses demonstrate that higher education was related to better self-rated health and fewer health events, fewer musculoskeletal symptoms and fewer mental health problems (the latter mainly driven by adjusting for age). Notably, educational disparities were more pronounced about musculoskeletal symptoms compared to mental health problems. Further analysis reveals that lower back pain and pain in the neck and shoulder area were the most prevalent musculoskeletal symptoms among both groups (each affecting approximately one-third of the sample), while general tiredness, faintness, and fatigue were the most common mental health problems (affecting over 40 percent) (see Supplementary material, eFig 2). Each of the musculoskeletal and mental health symptoms was more prevalent among workers with lower levels of education.

**Table 2:** Occupational health in relation to education, job demands and company characteristics

	Self-rated health		Number of health events		Number of musculoskeletal symptoms		Number of mental health problems	
	b [95% CI]		RR [95% CI]		RR [95% CI]		RR [95% CI]	
	Crude	M1	Crude	M1	Crude	M1	Crude	M1
School education								
High versus low	0.18***	0.24***	0.72***	0.74***	0.74***	0.73***	0.94	0.84***
	[0.12,0.24]	[0.18,0.31]	[0.66,0.79]	[0.67,0.82]	[0.69,0.80]	[0.66,0.80]	[0.86,1.03]	[0.76,0.93]
Job demands								
Physical job demands (Std)	− 0.06***	− 0.10***	1.20***	1.23***	1.34***	1.48***	1.12***	1.21***
	[− 0.09,− 0.03]	[− 0.13,− 0.07]	[1.16,1.25]	[1.18,1.28]	[1.30,1.39]	[1.43,1.53]	[1.07,1.16]	[1.16,1.27]
Psychosocial job demands (Std)	− 0.15***	− 0.15***	1.26***	1.22***	1.32***	1.32***	1.43***	1.42***
	[− 0.18,− 0.12]	[− 0.17,− 0.12]	[1.22,1.31]	[1.18,1.27]	[1.28,1.36]	[1.28,1.36]	[1.39,1.48]	[1.37,1.46]
Total job demands (Std)	− 0.13***	− 0.15***	1.29***	1.27***	1.41***	1.47***	1.35***	1.40***
	[− 0.16,− 0.10]	[− 0.18,− 0.12]	[1.24,1.34]	[1.23,1.32]	[1.37,1.46]	[1.43,1.52]	[1.31,1.40]	[1.36,1.45]
Company characteristics								
Company size (Std)	0.05**	0.03*	0.97	0.96	0.86***	0.87***	0.99	1.00
	[0.02,0.08]	[0.00,0.06]	[0.93,1.01]	[0.92,1.00]	[0.83,0.90]	[0.84,0.91]	[0.95,1.03]	[0.96,1.05]
Workplace health promotion	0.11***	0.10**	0.90*	0.86***	0.76***	0.77***	0.79***	0.78***
	[0.05,0.16]	[0.04,0.16]	[0.83,0.99]	[0.79,0.94]	[0.70,0.82]	[0.71,0.83]	[0.72,0.86]	[0.72,0.86]
Company in poor economic situation	− 0.18*	− 0.17*	1.28**	1.28**	1.18*	1.16	1.43***	1.46***
	[− 0.31,− 0.04]	[− 0.30, 0.04]	[1.08,1.51]	[1.09,1.51]	[1.00,1.38]	[0.99,1.36]	[1.21,1.70]	[1.24,1.73]
Downsizing in the past two years	− 0.11***	− 0.10**	1.35***	1.31***	1.20***	1.18***	1.41***	1.39***
	[− 0.17,− 0.05]	[− 0.16,− 0.03]	[1.23,1.47]	[1.20,1.43]	[1.11,1.30]	[1.09,1.28]	[1.29,1.54]	[1.28,1.52]

Data: BIBB/BAuA Youth Employment Survey 2012. *n* = 3142. Unstandardised linear regression coefficients (b) for self-rated health (1 = poor to 5 = excellent). Rate Ratios (RR) for health indicators with count character (number of health events, number of musculoskeletal symptoms, number of mental problems). Regression analysis separately conducted for each predictor variable. M1 = Adjusted for age (one-year increments), sex, nationality, region, employment relation, weekly working hours, job tenure, working sector. Continuous variables were standardised (Std) for better comparison of effect estimates

*CI*   confidence interval

*p < 0.05, **p < 0.01, ***p < 0.001

Furthermore, higher job demands were linked to adverse health outcomes. Interestingly, physical job demands exhibited a stronger association with musculoskeletal symptoms compared to psychosocial job demands. In contrast, mental health symptoms displayed stronger associations with psychosocial job demands rather than physical ones. Table 2 also reveals that company characteristics were associated with different health outcomes. Workers in larger companies reported better general health and lower numbers of musculoskeletal symptoms compared to those in smaller companies. However, there were no variations found regarding mental health issues and other health events between different company sizes. The relationship between company characteristics and workers’ health was more consistent for other factors studied. Specifically, workplace health promotion programs were linked to better general health and lower numbers of health events and physical and mental health problems. In contrast, situations involving downsizing or economic hardships within a company were related to worse self-rated health and a higher prevalence of musculoskeletal and mental health symptoms.

**Fig. 2:**
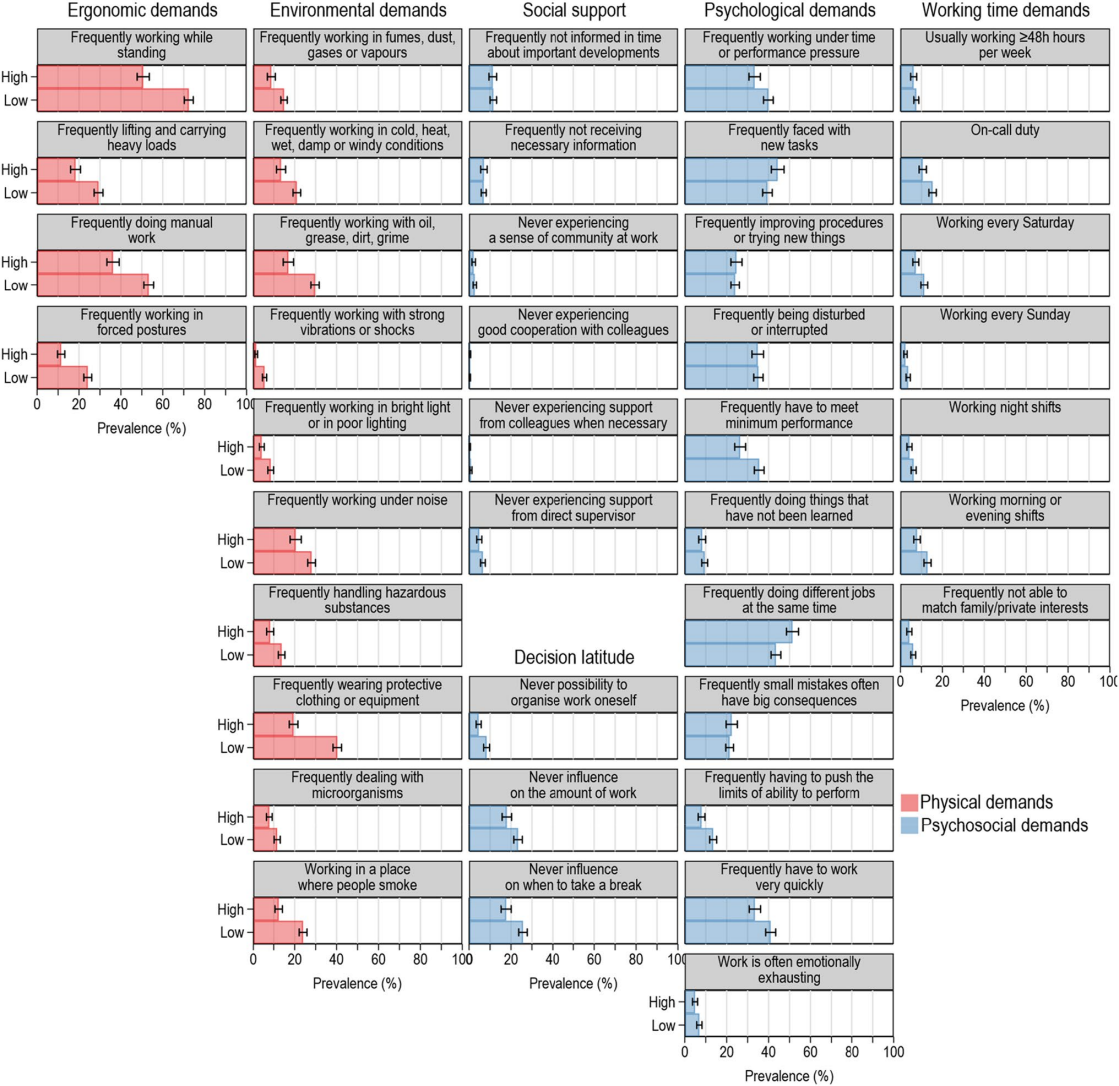
Prevalence of job demands by low (< 12 years) and high (≥ 12 years) levels of school education. Data: BIBB/BAuA Youth Employment Survey 2012. *n* = 3142. Relative frequencies with 95% confidence intervals. Estimates were adjusted for age, sex, nationality, region, employment relation, weekly working hours, job tenure, and working sector

Figure 2 displays the prevalence of single job demands based on workers' educational attainment. Workers with lower levels of education had a higher prevalence for all 14 indicators of physical demands examined. On the other hand, not all psychosocial demands showed a pattern associated with education. Although a lack of decision-making autonomy and working time requirements were more prevalent among workers with less education, indicators for low social support or high psychological demands did not consistently align with this pattern.

### Mediation analysis

Results of the mediation analysis are presented in Table 3. Overall, both physical and psychosocial job demands (and their combination) showed significant indirect effects and mediated the statistical association between high education and health. In general, the percentage mediated was higher for musculoskeletal and mental health symptoms compared with self-rated health and health events. Furthermore, physical job demands explained a larger part of musculoskeletal disorders compared with mental health problems.

**Table 3:** Indirect effects of high education on health outcomes via job demands

	Self-rated health			Number of health events			Number of musculoskeletal symptoms			Number of mental health problems		
Mediator	Est	95% CI		Est	95% CI		Est	95% CI		Est	95% CI	
Physical job demands												
Indirect effect	**0.036**	**0.020**	**0.053**	**− 0.798**	**− 1.068**	**− 0.567**	**− 0.258**	**− 0.315**	**− 0.208**	**− 0.090**	**− 0.122**	**− 0.064**
Direct effect	0.211	0.143	0.276	− 2.112	− 3.048	− 1.224	− 0.214	− 0.339	− 0.095	− 0.103	− 0.217	0.005
Total effect	0.247	0.179	0.310	− 2.910	− 3.885	− 2.014	− 0.471	− 0.599	− 0.347	− 0.193	− 0.309	− 0.087
% mediated	14.5%	11.6%	20.0%	27.6%	20.5%	39.6%	54.9%	43.0%	74.3%	47.2%	29.3%	103.6%
Psychosocial job demands												
Indirect effect	**0.029**	**0.016**	**0.043**	**− 0.446**	**− 0.658**	**− 0.268**	**− 0.098**	**− 0.139**	**− 0.061**	**− 0.101**	**− 0.142**	**− 0.064**
Direct effect	0.218	0.150	0.282	− 2.466	− 3.399	− 1.580	− 0.373	− 0.497	− 0.255	− 0.093	− 0.198	0.007
Total effect	0.247	0.179	0.310	− 2.912	− 3.874	− 2.002	− 0.471	− 0.598	− 0.345	− 0.194	− 0.303	− 0.085
% mediated	11.8%	9.4%	16.3%	15.3%	11.5%	22.3%	20.9%	16.4%	28.5%	52.2%	33.4%	119.2%
Total job demands												
Indirect effect	**0.054**	**0.037**	**0.070**	**− 0.944**	**− 1.234**	**− 0.710**	**− 0.250**	**− 0.310**	**− 0.197**	**− 0.168**	**− 0.211**	**− 0.131**
Direct effect	0.194	0.126	0.258	− 1.968	− 2.894	− 1.089	− 0.222	− 0.342	− 0.108	− 0.026	− 0.135	0.078
Total effect	0.247	0.181	0.310	− 2.912	− 3.891	− 1.995	− 0.472	− 0.600	− 0.349	− 0.194	− 0.304	− 0.087
% mediated	21.6%	17.3%	29.6%	32.5%	24.3%	47.3%	53.1%	41.7%	71.6%	87.2%	55.3%	192.9%

Data: BIBB/BAuA Youth Employment Survey 2012. *n* = 3142. Estimates in boldface indicate statistical significance of the indirect effect. Positive effects indicate better self-rated health or more numbers of health events or symptoms, while negative effects indicate lower self-rated health and lower numbers of health events or symptoms

Table 4 shows that company size mediated the association between education and musculoskeletal disorders, but did not reveal significant mediation in terms of other health outcomes. Workplace health promotion was a significant mediator for the association between education and all health outcomes. A poor economic situation of the company and downsizing did not show significant mediation effects. Mediation effects of company characteristics, if significant, were smaller compared with individual job demands.

**Table 4:** Indirect effects of high education on health outcomes via company characteristics

	Self-rated health			Number of health events			Number of musculoskeletal symptoms			Number of mental health problems		
Mediator	Est	95% CI		Est	95% CI		Est	95% CI		Est	95% CI	
Company size												
Indirect effect	0.006	− 0.003	0.016	− 0.065	− 0.196	0.063	**− 0.046**	**− 0.067**	**− 0.028**	0.004	− 0.012	0.019
Direct effect	0.240	0.171	0.306	− 2.840	− 3.796	− 1.932	− 0.423	− 0.557	− 0.296	− 0.197	− 0.310	− 0.089
Total effect	0.247	0.179	0.313	− 2.905	− 3.841	− 1.991	− 0.470	− 0.598	− 0.343	− 0.193	− 0.303	− 0.085
% mediated	N/A			N/A			9.9%	7.8%	13.5%	N/A		
Workplace health promotion												
Indirect effect	**0.006**	**0.001**	**0.013**	**− 0.099**	**− 0.186**	**− 0.028**	**− 0.027**	**− 0.044**	**− 0.012**	**− 0.020**	**− 0.032**	**− 0.008**
Direct effect	0.239	0.171	0.304	− 2.791	− 3.739	− 1.890	− 0.438	− 0.569	− 0.314	− 0.170	− 0.281	− 0.064
Total effect	0.246	0.179	0.312	− 2.889	− 3.809	− 1.979	− 0.465	− 0.592	− 0.341	− 0.190	− 0.297	− 0.083
% mediated	2.6%	2.1%	3.6%	3.4%	2.6%	5.0%	5.8%	4.6%	8.0%	10.5%	6.6%	23.7%
Company in poor economic situation												
Indirect effect	− 0.001	− 0.006	0.003	0.013	− 0.055	0.095	0.001	− 0.006	0.010	0.002	− 0.010	0.016
Direct effect	0.248	0.180	0.313	− 2.930	− 3.874	− 2.034	− 0.472	− 0.603	− 0.348	− 0.197	− 0.308	− 0.092
Total effect	0.247	0.180	0.311	− 2.917	− 3.855	− 2.004	− 0.471	− 0.601	− 0.344	− 0.195	− 0.304	− 0.088
% mediated	N/A			N/A			N/A			N/A		
Downsizing												
Indirect effect	0.002	− 0.001	0.007	− 0.063	− 0.170	0.035	− 0.006	− 0.017	0.003	− 0.009	− 0.025	0.005
Direct effect	0.245	0.177	0.310	− 2.866	− 3.812	− 1.969	− 0.466	− 0.597	− 0.342	− 0.187	− 0.297	− 0.082
Total effect	0.247	0.180	0.312	− 2.930	− 3.887	− 2.020	− 0.472	− 0.604	− 0.347	− 0.196	− 0.308	− 0.089
% mediated	N/A			N/A			N/A			N/A		

Data: BIBB/BAuA Youth Employment Survey 2012. *n* = 3142. Estimates in boldface indicate statistical significance of the indirect effect. Positive effects indicate better self-rated health or more numbers of health events or symptoms, while negative effects indicate lower self-rated health and lower numbers of health events or symptoms. Percentage mediated not assigned (N/A) in case of insignificant indirect effect estimate

## Discussion

Findings of this study provide evidence for the existence of pronounced socio-economic disparities in occupational health among young workers in Germany. Educational gradients were observed for self-rated health, as well as for the number of health events, symptoms of musculoskeletal disorders, and symptoms of mental health problems over the last 12 months. Moreover, based on the results of the mediation analyses, we conclude that the socio-economic gradient is explained by higher physical and psychosocial job demands of low educated workers, although physical demands mediated a greater part of the health inequalities (except for mental health problems). In addition, more unfavourable company characteristics (working in small companies with lacking health promotion measures, financial strains and downsizing) accounted less often and to a smaller degree for health inequalities compared with individual job demands.

In general, our findings suggest that the extent and causes of occupational health disparities in young workers are quite comparable to what has been observed in studies based on samples of middle-aged and older workers (Muntaner et al. 2004; Hansen and Andersen 2008; Christensen et al. 2008; Hoven and Siegrist 2013; Lund et al. 2018; Dieker et al. 2019; Perhoniemi et al. 2020; Karran et al. 2020). Furthermore, our findings confirm those obtained in two previous studies that examined occupational health inequalities among young workers. A study based on Canadian workers aged 20–29 years found that job demands explained educational inequalities in self-rated health by 12.5% (Karmakar and Breslin 2008). Another study based on Finnish employees between the ages of 25–34 years observed an association between low education and higher sickness absence rates (without employing mediation analysis) (Sumanen et al. 2015). Our study confirms these findings, not only within the German context but also in a younger age group (15–24 years), utilising more precise indicators of health such as musculoskeletal and mental health symptoms.

As we controlled in our analyses for the working sector and the employment relationship, we argue that findings may not be explained by selection processes alone (i.e., low school education selects in manual jobs with physical demands) (Erikson and Goldthorpe 1992; Ravesteijn et al. 2013). Another possible explanation could be that low-educated workers are more inclined to accept unfavourable working conditions due to limited job opportunities (Landsbergis et al. 2014) or a lack of negotiating power. Supporting this explanation, our study also revealed disparities in psychosocial job demands, particularly in terms of working time arrangements (on-call duty, weekend work, and shift work frequency) and decision autonomy (i.e., lack of influence on the organisation and amount of work). The latter is likely to be explained by the fact that individuals with lower formal qualifications often hold lower positions in the occupational status hierarchy.

The results of our study are concerning because early health disadvantages pose a particular risk for manifestation over the remaining life course (Ben-Shlomo and Kuh 2002; Ferraro et al. 2009). This is due to two distinct processes of risk accumulation which connect youth health inequalities with future health outcomes. First, individuals with lower education levels are more likely to be exposed to hazardous working conditions from the beginning of their careers, resulting in a longer cumulative duration of work exposure compared to those who are better educated. This extended exposure may contribute towards socio-economic disparities in work ability and premature labour market exit later in life (Hasselhorn 2020). Second, inequalities in health problems early in life often have important consequences for subsequent life stages, because initial health problems are likely to be followed by additional disadvantages. For example, persistent musculoskeletal symptoms like back pain or knee pain can diminish one's ability to work and result in job loss. These experiences can also contribute to deteriorating health through chronic stress, adopting unhealthy behaviours, or facing material hardships. Likewise, mental health problems frequently raise the likelihood of unemployment due to reduced productivity and increased rates of absenteeism.

From our perspective, there are several potential strategies to address these problems. Firstly, companies can prioritise enhanced workplace safety and health measures, particularly for young workers, by addressing differing workloads such as ergonomic demands (e.g., malposition when lifting and carrying heavy loads) and environmental workplace risks (e.g., better protection against dusts, gases, noise, or hazardous substances). Support at a governmental level could also be beneficial, such as through improved regulations on occupational health and safety and mandatory inspections within companies. Nonetheless, it is important to acknowledge that reducing health inequalities remains a challenge and few studies have examined the effectiveness of various public health approaches in mitigating these disparities in the workplace context (Thomson et al. 2018). However, our research highlights the importance of considering early career stages when stratification processes can still be modified.

When considering the findings of our study on young workers in Germany, it is important to acknowledge certain aspects related to the regional education and labour market system before generalising these results to other countries. Specifically, Germany's emphasis on standardised vocational educational training programs enhances the employability of young workers and reduces their risk for unemployment (Bol and Werfhorst 2013). As a result, the relationship between education level and health outcomes may be less pronounced in countries with higher rates of youth unemployment since individuals with lower levels of education are more likely to be excluded from the workforce. Moreover, as shown by the 2015 European Working Conditions Survey, Germany has an above-average score in terms of physical work hazards when compared to other European countries (Eurofound 2017). Consequently, the high proportion of health inequalities that was mediated by physical demands could be attributed to the greater proportion of workers in Germany who are exposed to such hazards compared with other nations.

### Strengths and limitations

Due to the cross-sectional study design, we were not able to establish a causal order between most variables and, thus, cannot preclude that initial health problems could also have changed job demands (due to job change or response bias). Another limitation is that we could not consider other mediators of relevance for young workers, particularly precarious and insecure employment relations. However, due to the German vocational training system, we found assessments of objective (temporary contract) and subjective job insecurity (perceived likelihood of job loss) highly inflated by voluntary forms of job terminations (e.g., ending of apprenticeship programs or student jobs). Another limitation could be that interviews were only conducted in German and other interview languages were not considered, which may have excluded social groups with high vulnerability (e.g., migrant workers). Furthermore, it is worth considering that the results obtained from the data collected in 2012 may not fully reflect the experiences of younger cohorts of workers today. Changes over time, such as a decrease in smoking prevalence and improved occupational safety measures, could potentially lead to fewer workers being exposed to some of the workplace hazards (e.g., second-hand smoking). Conversely, certain job demands like heat exposure might have become even more prevalent in recent years. However, by utilising a broad set of physical and psychosocial job demands, we argue that most of the indicators still reflect common workplace experiences in Western workforces.

A strength of our study is the use of a youth employment survey located at an often-overlooked life stage, namely the very early labour market career (15–24 years), where not only employees but also apprentices, trainees and student workers were recruited. Yet most evidence for the extent of health inequalities in this life stage stemmed from youth cohort studies (Quon and McGrath 2014; Sweeting et al. 2015), which are not based on workforce samples and, thus, include large parts of economic inactive people (e.g., students or unemployed persons). A further strength was that the socio-economic position was operationalised according to school education, which is generally completed before the labour market entry. Thus, despite using a cross-sectional design, this allowed for establishing a temporal order and minimising reversal causation bias. Another strength is that we could include a rich number of occupation health indicators, while controlling for important socio-demographic and occupational covariates.

## Conclusions

Our findings suggest that educational inequalities in occupational health are already present among young workers and mediated by physical and psychosocial job demands. In addition, variations in workplace health promotion measures at the company-level account for some part of educational health inequalities (although to a lesser extent than individual working conditions). We suggest that prevention measures that aim to tackle occupational health inequalities could be improved by shifting the focus on addressing socio-economically stratified job demands in earlier life stages.

### Supplementary Information

Below is the link to the electronic supplementary material.

Supplementary file1 (DOCX 380 KB)

## Data Availability

This study uses data of the BIBB/BAuA Youth Employment Survey 2012 (Jugenderwerbstätigenbefragung, J-ETB), a study conducted by the Federal Institute for Vocational Education and Training (BIBB) and the German Federal Institute for Occupational Safety and Health (BAuA). The dataset analyzed during the current study is available in the BIBB Research Data Centre repository (https://www.bibb.de/en/53.php).
