# Supplementary material for: Health inequalities among young workers: the mediating role of working conditions and company characteristics
Source: Int Arch Occup Environ Health. 2023 Oct 9;96(10):1313–24. doi: 10.1007/s00420-023-02010-6 (PMC10635983; doi:10.1007/s00420-023-02010-6)
Supplement: Supplementary file 1 — Supplementary file1 (DOCX 380 KB) [file 420_2023_2010_MOESM1_ESM.docx]

# Supplementary Information

**Article:** Health inequalities among young workers: The mediating role of working conditions and company characteristics

**Journal:** International Archives of Occupational and Environmental Health

**Authors:** Marvin Reuter, Claudia R Pischke, Mariann Rigo, Katharina Diehl, Jacob Spallek, Matthias Richter, Claudia Hövener, Nico Dragano

**Corresponding author:** Marvin Reuter, Junior Professorship for Sociology, esp. Work and Health, University of Bamberg, Feldkirchenstraße 21, 96045 Bamberg, Germany, Mail: [marvin.reuter@uni-bamberg.de](mailto:marvin.reuter@uni-bamberg.de)

**e-Table 1:** Assessment of health problems at work during the last 12 months.

| **No** | **Symptoms of musculoskeletal disorders** | **No** | **Symptoms of mental health problems** |
| --- | --- | --- | --- |
| 1 | Lower back pain | 1 | Sleep disturbances at night |
| 2 | Pain in the neck, shoulder area | 2 | General tiredness, faintness or fatigue |
| 3 | Pain in the arms | 3 | Nervousness or irritability |
| 4 | Pain in the hands | 4 | Low mood |
| 5 | Pain in the hips | 5 | Emotional exhaustion |
| 6 | Pain in the knees |  |  |
| 7 | Pain in the legs, feet |  |  |

Original question: “Please tell me if you have experienced the following health complaints during the last 12 months at work. We are interested in the complaints that occurred frequently.”

**e-Table 2:** Indicators of physical and psychosocial job demands (**α**=Cronbach’s alpha)

|  |  | **α** | **Mean** | **SD** | **n** |
| --- | --- | --- | --- | --- | --- |
| **Physical demands (Ergo + Env)** | | **0.81** | **3.22** | **2.92** | **3,142** |
| **Ergonomic demands (Ergo)** | | 0.66 | 1.56 | 1.28 | 3,142 |
|  | (1) Working while standing |  |  |  |  |
|  | (2) Lifting and carrying heavy loads (Men ≥20 kg, women ≥10 kg) |  |  |  |  |
|  | (3) Manual work requiring great dexterity, fast sequences of movements or greater strength |  |  |  |  |
|  | (4) Working in forced postures |  |  |  |  |
| **Environmental demands (Env)** | | 0.73 | 1.67 | 1.96 | 3,142 |
|  | (1) Work in fumes, dust, gases or vapours |  |  |  |  |
|  | (2) Work in cold, heat, wet, damp or windy conditions |  |  |  |  |
|  | (3) Working with oil, grease, dirt, grime |  |  |  |  |
|  | (4) Working with strong vibrations, shocks and oscillations that are felt in the body |  |  |  |  |
|  | (5) Work in bright light or in poor or too dim lighting |  |  |  |  |
|  | (6) Working under noise |  |  |  |  |
|  | (7) Handling hazardous substances |  |  |  |  |
|  | (8) Wearing protective clothing or equipment |  |  |  |  |
|  | (9) Dealing with microorganisms such as pathogens, bacteria, moulds or viruses |  |  |  |  |
|  | (10) Do you work in a place where people smoke? |  |  |  |  |
| **Psychosocial demands (Sup + Dec + Psy + Time)** | | **0.64** | **4.35** | **2.88** | **3,142** |
| **Low social support (Sup)** | | 0.36 | 0.28 | 0.60 | 3,142 |
|  | (1) Frequently not informed in time about important developments in the company |  |  |  |  |
|  | (2) Frequently not receiving necessary information |  |  |  |  |
|  | (3) Never experiencing a sense of community at work |  |  |  |  |
|  | (4) Never experiencing good cooperation with colleagues |  |  |  |  |
|  | (5) Never experiencing support from colleagues when necessary |  |  |  |  |
|  | (6) Never experiencing support from direct supervisor |  |  |  |  |
| **Low decision latitude (Dec)** | | 0.43 | 0.51 | 0.75 | 3,142 |
|  | (1) Never possibility to organise work oneself |  |  |  |  |
|  | (2) Never having influence on the assigned amount of work |  |  |  |  |
|  | (3) Never having influence on when to take a break |  |  |  |  |
| **Psychological demands (Psy)** | | 0.62 | 3.03 | 2.14 | 3,142 |
|  | (1) Frequently working under time or performance pressure |  |  |  |  |
|  | (2) Frequently faced with new tasks |  |  |  |  |
|  | (3) Frequently improving procedures or trying new things |  |  |  |  |
|  | (4) Frequently being disturbed/interrupted at work |  |  |  |  |
|  | (5) Frequently have to meet minimum performance |  |  |  |  |
|  | (6) Frequently doing things that have not been learned |  |  |  |  |
|  | (7) Frequently doing different jobs at the same time |  |  |  |  |
|  | (8) Frequently small mistakes often have big consequences |  |  |  |  |
|  | (9) Frequently having to push the limits of ability to perform |  |  |  |  |
|  | (10) Frequently have to work very quickly |  |  |  |  |
|  | (11) Work is often emotionally exhausting |  |  |  |  |
| **Working time demands (Time)** | | 0.44 | 0.54 | 0.89 | 3,142 |
|  | (1) Usual working hours ≥48h per week (including overwork) |  |  |  |  |
|  | (2) On-call duty |  |  |  |  |
|  | (3) Working every Saturday |  |  |  |  |
|  | (4) Working every Sunday |  |  |  |  |
|  | (5) Working night shifts |  |  |  |  |
|  | (6) Working morning or evening shifts |  |  |  |  |
|  | (7) Frequently not able to match family/private interests |  |  |  |  |
| **Total score: Working demands (Physical + Psychosocial)** | | **0.78** | **7.58** | **4.74** | **3,142** |

**e-Table 3:** Missing information before imputation

| **Variable** |  | **Missing (n)** | **Valid (n)** | **Categories** | **Min** | **Max** |
| --- | --- | --- | --- | --- | --- | --- |
| sex | Sex |  | 3,214 | 2 | 1 | 2 |
| age | Age |  | 3,214 | 10 | 15 | 24 |
| nation | Nationality |  | 3,214 | 2 | 1 | 2 |
| region | Region |  | 3,214 | 2 | 1 | 2 |
| mscdis | Symptoms of musculoskeletal disorders | | 3,214 | 10 | 7 | 63 |
| mentdis | Symptoms of mental disorders |  | 3,214 | 9 | 5 | 31 |
| physical | Physical job demands |  | 3,214 | 15 | 0 | 14 |
| psychosocial | Psychosocial job demands |  | 3,214 | 21 | 0 | 21 |
| wrkh | Weekly working hours | 2 | 3,212 | 103 | 1 | 84 |
| jobten | Job tenure | 18 | 3,196 | 11 | 0 | 11 |
| edu | Education | 39 | 3,175 | 4 | 9 | 13 |
| empst | Employment status | 5 | 3,209 | 4 | 1 | 4 |
| compsize | Company size | 224 | 2,990 | 11 | 1 | 1000 |
| wrksec | Working sector | 138 | 3,076 | 10 | 1 | 10 |
| econ | Company economic situation | 188 | 3,026 | 2 | 0 | 1 |
| whp | Company health promotion | 390 | 2,824 | 2 | 0 | 1 |
| downsizing | Company downsizing | 84 | 3,130 | 2 | 0 | 1 |
| srhlth | Self-rated health | 2 | 3,212 | 5 | 1 | 5 |
| absent_days | Absenteeism days | 26 | 3,188 | 56 | 0 | 365 |
| present_days | Presenteeism days | 68 | 3,146 | 40 | 0 | 200 |
| *parocc* | *Parental occupational class* | 185 | 3,029 | 4 | 1 | 4 |
| *eseg* | *European socio-economic group* | 58 | 3,156 | 7 | 1 | 7 |
| *income* | *Job income* | 288 | 2,926 | 432 | 1 | 21000 |

Variables marked in italics were used as auxiliary variables in the imputation model.

**e-Table 4:** Original versus imputed data set

|  | **Original** | | | | |  | **Imputed** | | | | |
| --- | --- | --- | --- | --- | --- | --- | --- | --- | --- | --- | --- |
|  | **n** | **Mean** | **SD** | **Min** | **Max** |  | **n** | **Mean** | **SD** | **Min** | **Max** |
| wrkh | 3,209 | 35.9 | 11.3 | 10 | 84 |  | 3,214 | 35.9 | 11.3 | 10 | 84 |
| jobten | 3,196 | 2.0 | 1.7 | 0 | 11 |  | 3,214 | 2.0 | 1.7 | 0 | 11 |
| edu | 3,175 | 11.0 | 1.5 | 9 | 13 |  | 3,214 | 11.0 | 1.5 | 9 | 13 |
| empst | 3,209 | 2.4 | 1.2 | 1 | 4 |  | 3,214 | 2.4 | 1.2 | 1 | 4 |
| compsize | 2,990 | 256.9 | 354.6 | 1 | 1000 |  | 3,214 | 252.4 | 352.4 | 1 | 1000 |
| wrksec | 3,076 | 5.7 | 2.7 | 1 | 10 |  | 3,214 | 5.7 | 2.7 | 1 | 10 |
| econ | 3,026 | 0.1 | 0.2 | 0 | 1 |  | 3,214 | 0.1 | 0.2 | 0 | 1 |
| whp | 2,824 | 0.5 | 0.5 | 0 | 1 |  | 3,214 | 0.4 | 0.5 | 0 | 1 |
| downsizing | 3,130 | 0.3 | 0.5 | 0 | 1 |  | 3,214 | 0.3 | 0.5 | 0 | 1 |
| srhlth | 3,212 | 3.6 | 0.8 | 1 | 5 |  | 3,214 | 3.6 | 0.8 | 1 | 5 |
| absent_days | 3,188 | 6.4 | 15.0 | 0 | 365 |  | 3,214 | 6.4 | 15.0 | 0 | 365 |
| present_days | 3,146 | 4.9 | 10.7 | 0 | 200 |  | 3,214 | 5.0 | 10.7 | 0 | 200 |


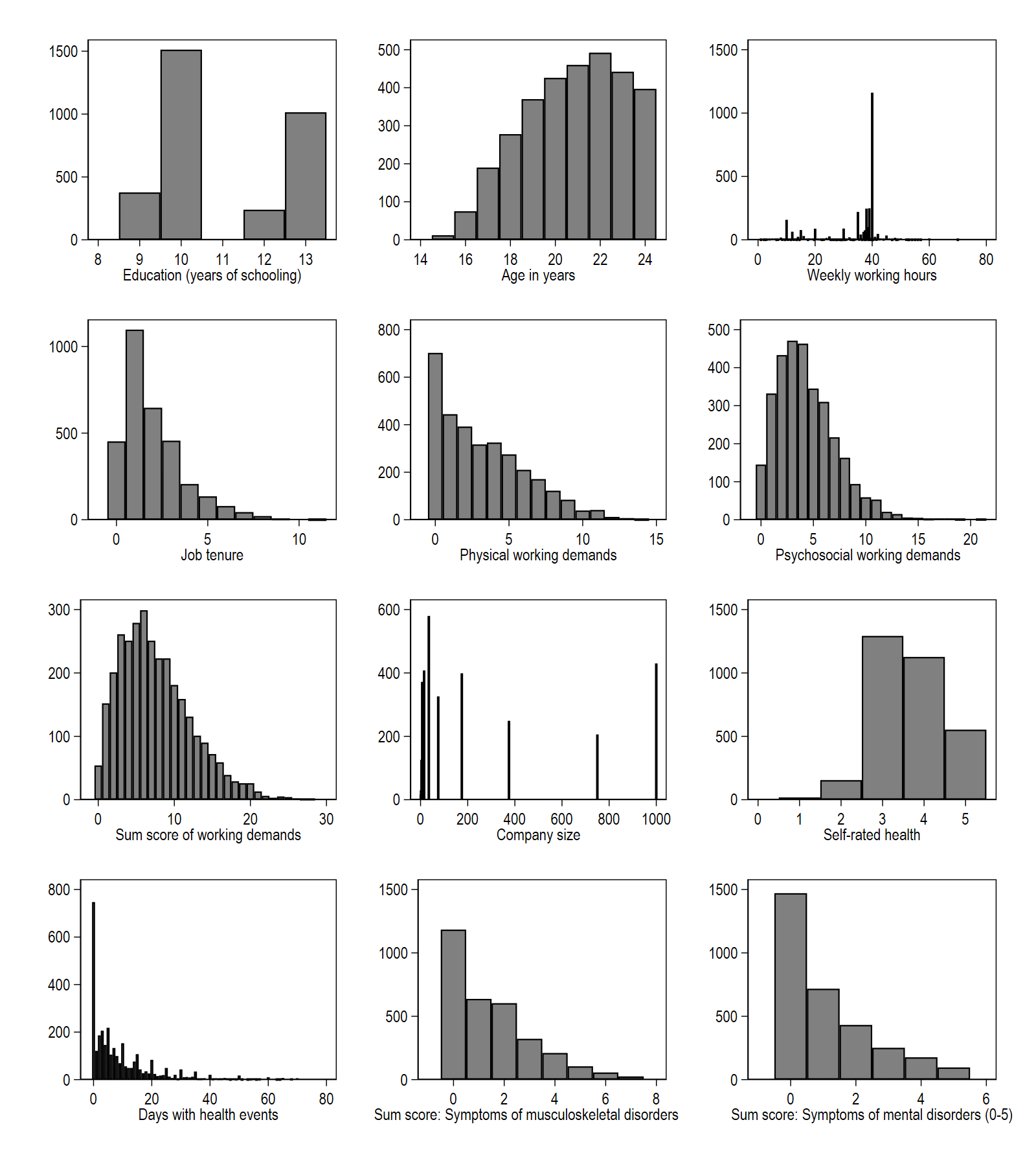


**eFig 1** Histogram illustrating the distribution of continuous variables. n=3,142.


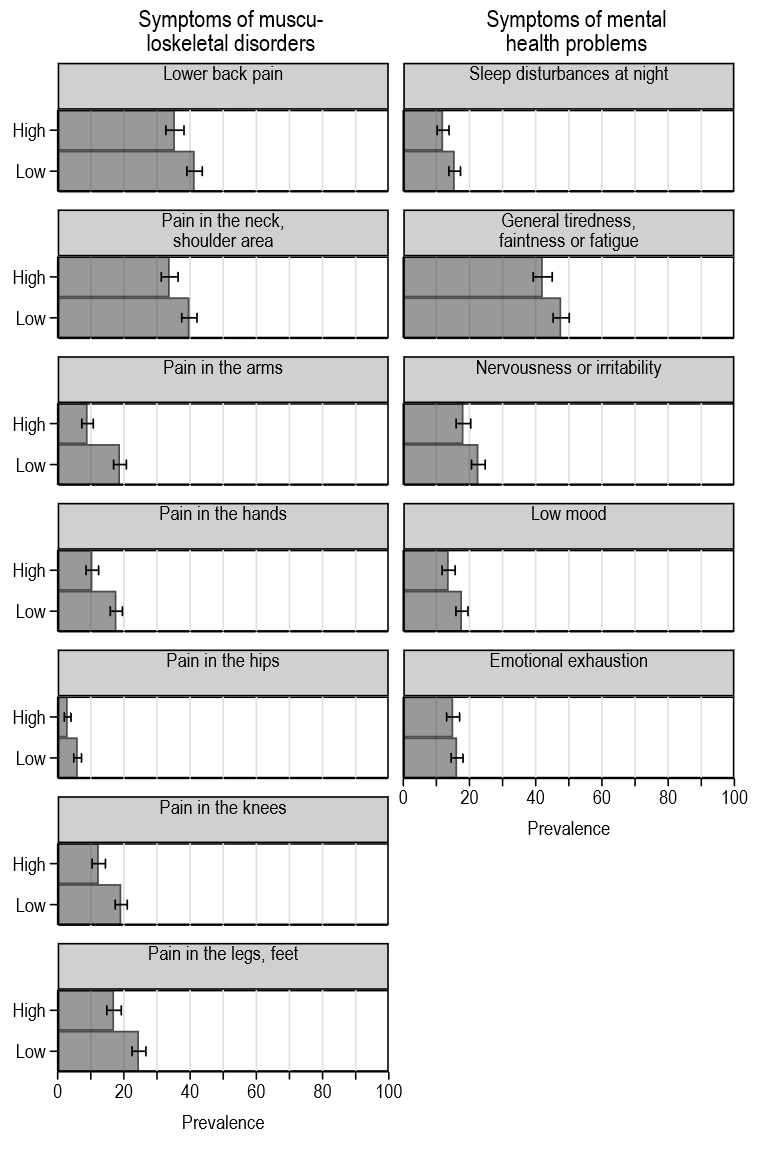


**eFig 2** Prevalence (%) of musculoskeletal symptoms and mental health problems by low (<12 years) and high (≥12 years) levels of school education

Data: BIBB/BAuA Youth Employment Survey 2012. n=3,142. Relative frequencies with 95% confidence intervals. Estimates were adjusted for age, sex, nationality, region, employment relation, weekly working hours, job tenure, and working sector.
